# Parental perception about the pandemic impact on mental health of children: a cross-sectional study

**DOI:** 10.1093/eurpub/ckac130.236

**Published:** 2022-10-25

**Authors:** S Pinto, G Lo Moro, F Bert, E Rolfini, G Scaioli, R Siliquini

**Affiliations:** 1 Department of Public Health Sciences, Università degli Studi di Torino, Turin, Italy; 2 A.O.U. City of Health and Science of Turin, Università degli Studi di Torino, Turin, Italy

## Abstract

**Background:**

The pandemic may increase mental issues among children. This study aimed to explore parental perceptions on the pandemic impact on the health status of their children, with a focus on mental health.

**Methods:**

An online nationwide cross-sectional study has been conducted amongst Italian parents (from April 2022-ongoing). The survey included: Strength and Difficulties Questionnaire (SDQ), Kessler-6 (K6) for parent's psychological distress, and pandemic-related items. The outcomes were: child's SDQ above the clinical cut-off and perceived child's worsening of sleep, appetite, physical and mental health during the pandemic. Multivariable regressions were run (p < 0.05 as significant).

**Results:**

Up to date, participants were 333 (88% female). Mean age was 40.7 years (SD = 6.7). Considering their children, 52.9% were female and mean age was 6.62 (SD = 4.3). A total of 12.6% of children passed the SDQ cut-off. Having parents who are healthcare workers (adjOR=4.1), having parents positive for K6 (adjOR=4.0) and having a poor economic situation (adjOR=3.9) were significantly associated with a higher probability of passing the cut-off. Considering the pandemic, 15.4% declared their child had worse sleep, 12.2% lower appetite, 6.6% more physical issues, and 22.9% more mental issues. Using electronic devices more than before the pandemic was significantly associated with worsening of sleep (adjOR=2.9) and appetite (adjOR=6.9). Having parents who are healthcare workers was significantly associated with worsening of sleep (adjOR=2.3) and mental health (adjOR=2.4). Having parents positive for K6 was significantly associated with worsening of mental health (adjOR=5.3).

**Conclusions:**

This study suggested a perceived substantial worsening of children's health, especially considering mental health. Exploring how parents recognize their children's health and how the COVID-19 has changed daily habits should be considered as a public health priority in Europe.

**Key messages:**

- Children having parents who are healthcare workers, having parents positive for K6 and having a poor economic situation had a higher risk to pass the clinical cut-off of SDQ.
- According to parents’ perceptions, the impact of the pandemic was relevant on children's health, especially on mental health.

